# Competing risk survival analysis of time to in-hospital death or discharge in a large urban neonatal unit in Kenya

**DOI:** 10.12688/wellcomeopenres.15302.1

**Published:** 2019-06-17

**Authors:** Jalemba Aluvaala, Gary S. Collins, Beth Maina, Catherine Mutinda, Mary Wayiego, James A. Berkley, Mike English

**Affiliations:** 1 Paediatrics and Child Health, University of Nairobi, Nairobi, Kenya; 2 Centre for Tropical Medicine and Global Health, Nuffield Department of Medicine, University of Oxford, Oxford, UK; 3 KEMRI-Wellcome Trust Research Programme, Nairobi, Kenya; 4 Centre for Statistics in Medicine, Nuffield Department of Orthopaedics, Rheumatology and Musculoskeletal Sciences, Botnar Research Centre, University of Oxford, Oxford, UK; 5 Oxford University Hospitals NHS Foundation Trust, Oxford, UK; 6 Neonatal Unit, Pumwani Maternity Hospital, Nairobi, Kenya; 7 The Childhood Acute Illness & Nutrition (CHAIN) Network, Nairobi, Kenya

**Keywords:** Neonatal, mortality, survival, prognosis, hospital, Kenya, competing risks

## Abstract

**Background: **Clinical outcomes data are a crucial component of efforts to improve health systems globally. Strengthening of these health systems is essential if the Sustainable Development Goals (SDG) are to be achieved. Target 3.2 of SDG Goal 3 is to end preventable deaths and reduce neonatal mortality to 12 per 1,000 or lower by 2030. There is a paucity of data on neonatal in-hospital mortality in Kenya that is poorly captured in the existing health information system. Better measurement of neonatal mortality in facilities may help promote improvements in the quality of health care that will be important to achieving SDG 3 in countries such as Kenya.

**Methods: **This was a cohort study using routinely collected data from a large urban neonatal unit in Nairobi, Kenya. All the patients admitted to the unit between April 2014 to December 2015 were included. Clinical characteristics are summarised descriptively, while the competing risk method was used to estimate the probability of in-hospital mortality considering discharge alive as the competing risk.

**Results: **A total of 9,115 patients were included. Most were males (966/9115, 55%) and the majority (6287/9115, 69%) had normal birthweight (2.5 to 4 kg). Median length of stay was 2 days (range, 0 to 98 days) while crude mortality was 9.2% (839/9115). The probability of in-hospital death was higher than discharge alive for birthweight less than 1.5 kg with the transition to higher probability of discharge alive observed after the first week in birthweight 1.5 to <2 kg.

**Conclusions:** These prognostic data may inform decision making, e.g. in the organisation of neonatal in-patient service delivery to improve the quality of care. More of such data are therefore required from neonatal units in Kenya and other low resources settings especially as more advanced neonatal care is scaled up.

## Introduction

Clinical outcomes data are a crucial component of efforts to develop and improve health systems globally ^1^. Strengthening of these health systems is essential if the Sustainable Development Goals (SDG) are to be achieved ^2^. For neonates, Target 3.2 of SDG Goal 3 is to end preventable deaths and reduce mortality to 12 per 1,000 or lower by 2030 ^3^. In the Millennium Development Goal (MDG) era (2000–15) slower progress was observed in reduction of neonatal mortality relative to under 5 mortality. As a result, in 2015 neonatal mortality accounted for 45% of under 5 mortality in many countries, including Kenya ^4^. Going forward, better measurement of neonatal mortality in facilities may help promote improvements in the quality of health care that will be important to achieving SDG 3 in countries such as Kenya ^5, 6^.

There is, however, a paucity of data on neonatal in-hospital mortality in Kenya, as it is poorly captured in the existing health information system (District Health Information System version 2 (DHIS2)) ^7^. In 15 published reports over a 10-year period from Kenya (2007–16) that included approximately 20,000 neonates ( Table 1), the inpatient case fatality ratio for babies admitted to newborn care units (NBU, spanning all levels of dependency) varied markedly, ranging from 3 to 62 %. Only two reports included time to in-hospital death or length of stay ^8, 9^. These reports suggest a clear need for both better data capture and a need for more standardised approaches to reporting mortality from NBU.

**Table 1.** Neonatal inpatient case fatality in Kenyan hospitals.

Study	Year	Hospitals	Sample	Weight *	CFR
Were *et al.* ^14^	2007	1	344	<1500g	159/344 (46%)
Mwaniki *et al.* ^15^^‡^	2009	1	1105	All	336/1106 (30%)
Were *et al.* ^16^	2009	1	260	<1500g	116/260 (45%)
Talbert *et al.* ^17^^‡^	2010	1	Unspecified	All All	24% ^§^ vs 21% ^‖^ (0–6 days) 8% ^§^ vs 4% ^‖^ (7–28 days)
Mwaniki *et al.* ^18^^‡^	2010	1	1572 ^¶^	All	300/1572 (19%)
Mwaniki *et al.* ^8^^‡^	2010	1	8756	All	2053/8756 (24%) **
Mwaniki *et al.* ^19^^‡^	2010	1	5114	All	1011/5114 (20%)
Marete *et al.* ^20^	2011	1	135	<2500g	62/135 (46%)
Gathara *et al.* ^21^	2011	8	798	All	241/639 (38%)
Kohli-Kochhar ^22^	2011	1	152	Unspecified	4/152 (3%) ^††^
Yego *et al.* ^23^	2013	1	200	Unspecified	68/1000 live births
Aluvaala *et al.* ^9^	2015	22	1065	All	180/1065 (17%)
Ibinda *et al.* ^24^^‡^	2015	1	191	All	118/191 (62%) ^‡‡^
Aluvaala *et al.* ^25^	2015	5	1384	All	263/1384 (19%)
Myhre *et al.* ^26^	2016	1	118	<2500g	10/46(22%) vs 7/72 (11%) ^#^

*Birth weight eligible for inclusion in the study.

^‡^Same facility, different studies, part of a demographic surveillance system.

^§^All outborn, numbers not provided

^‖^All inborn, numbers not provided

^¶^Total neonatal admissions were 3302: excluded 1702 from outside DSS area and 28 readmissions

**Average over 19 years (1990–2008), reduced from 30.8% in 1990 to 16.5% in 2008

^††^Patients with blood culture positive sepsis only in a private tertiary hospital

^‡‡^Neonatal Tetanus only

^#^Before (22%) and after (11%) introduction of continuous positive airway pressure.

CFR, case report form.

Survival analysis approaches are well suited to examine both in-hospital mortality and length of stay to obtain insights beyond that provided by case fatality rates alone. The effect of competing risks, accounting of being discharged alive, must, however, be considered to avoid overestimation of the probability of the event of interest ^10, 11^. In neonatal survival analysis, Hinchliffe and colleagues demonstrated the utility of the competing risk approach for modelling length of stay where there are significant rates of mortality in the neonatal unit ^12^.

The prognostic data derived from such approaches may inform decision making in the organisation of neonatal in-patient service delivery to improve the quality of care ^13^. Other uses include more meaningful comparisons of mortality across multiple hospitals ^27^. For example, in the United Kingdom they have facilitated reorganisation of neonatal service provision by different levels of care and severity of illness ^28^. Our recent work in Nairobi, Kenya (a high mortality setting) suggests the need for strategic reorganisation of such services to improve quality of in-patient neonatal care. Utilisation data suggest 71% of available care is delivered in four public sector neonatal units but comparable outcome data are lacking and referral services are poorly developed ^29^. We therefore used routine data from the largest of the Nairobi facilities as a first step in better understanding in-hospital neonatal mortality and length of stay using competing risk survival analysis.

## Methods

### Study design

This was a retrospective cohort study using a routine inpatient data set from a large urban neonatal unit ^30^. Study participants were followed up from the time of admission to the unit to time of exit (defined as either death, discharge or referral).

### Setting

The study site was the neonatal unit in the largest public-sector maternity hospital in Nairobi, Kenya. The unit admits approximately 4500 neonates annually and on any given day has about 60 neonates cared for by 2-3 nurses per shift ^30^. Total medical staffing providing 24 hour 7 days a week care includes four Paediatricians, six Medical Officers and four Clinical Officers ^30^. The admitted neonates receive essential in-patient neonatal care with the most advanced intervention being limited capacity to provide continuous positive airway pressure (CPAP) therapy.

### Participants

All the patients admitted to the study neonatal unit between April 2014 to December 2015 were included. There were, however, no admissions between April to June 2015 due to industrial action.

### Variables

The key clinical characteristics are: sex, birthweight, mode of delivery, place of delivery, admission diagnosis and outcome. Birthweight was classified by the five typically used categories: extremely low birth weight (ELBW); <1 kg, very low birth weight (VLBW);1 to <1.5 kg, low birth weight (LBW); 1.5 to <2.5 kg, normal birth weight; 2.5 to 4 kg and macrosomia; >4 kg ^31^. The key variables in the survival analyses are outcome and time to exit from the unit measured in days. There were three possible outcomes: death, discharged alive and referred.

### Data sources and measurement

Data were abstracted from patient records at the point of exit from the unit and entered directly into the REDCap ^TM^ data capture tool that has been previously described ^32, 33^.

### Bias

Selection bias was minimised by including all patients admitted to the neonatal unit. In addition, data were entered immediately after discharge (or death) to reduce the chance of missing patient records. Observation bias was minimized by previously described data quality assurance procedures ^33^.

### Ethical approval

Ethical approval was provided by the KEMRI Scientific and Ethical Review Committee (SERU 3459). Individual consent was not required as de-identified data were abstracted from routine patient records after exit from the hospital to produce the secondary data used. The Kenya Ministry of Health gave permission for this work to be done.

### Statistical methods

Clinical characteristics are presented using descriptive statistics. Competing risks was used to estimate the probability of in-hospital mortality considering discharge alive as the competing risk using cumulative incidence functions ^34, 35^. The cumulative incidence functions were computed by estimating the joint probability of in-hospital mortality or discharge alive at a given time interval, given that the individual had not experienced either event in all prior intervals. The cumulative incidence at the end of a given time interval is the sum of the incidence in this interval and all previous intervals ^34^. Patients who were referred to other hospitals were right censored ^12^. These analyses were implemented in R (version 3.4.3) using the “cuminc” function of the “ cmprsk” package (version 2.2-7) ^35– 37^.

## Results

A total of 9,115 patients were included during the study period from April 2014 to December 2015. There were 5463 admissions in 2014 and 3652 in 2015. Admissions by month peaked at around 600 (June 2014), while the lowest number observed was around 250 (July 2015). The characteristics of the patients are presented in Table 2.

**Table 2.** Clinical characteristics of neonates included in the study data set.

Characteristic	All patients (n=9115)	
	n	%
Sex		
Male	4966	55
Missing	42	0.5
Birthweight (kg)		
<1	49	0.5
1 to <1.5	280	3
1.5 to <2.5	2132	23
2.5 to 4	6287	69
>4	353	4
Missing	23	0.3
Mode of delivery		
Spontaneous vaginal	5116	58
Assisted vaginal	4	0.04
Breech	90	1
Caesarean section	3660	41
Missing	245	3
Outborn		
Yes	226	3
Missing	0	
HIV exposure		
Exposed	547	6
Missing	529	6
Admission diagnosis *		
Birth asphyxia	3867	42
Preterm/LBW	2029	22
Neonatal sepsis	960	11
Respiratory distress syndrome	724	8
Neonatal jaundice	513	6
Others	1014	11
Outcome		
Discharged alive	8135	90
Dead	839	9
Referred	108	1
Missing	33	0.4
Mortality by birthweight (kg)		
Extremely low (<1)	45/49	92
Very low (1 to <1.5)	192/280	69
Low (1.5 to <2)	157/670	23
Low (2 to <2.5)	100/1448	7
Normal (2.5 to 4)	336/6253	5
Macrosomia (>4)	7/352	2
Length of stay by birth weight (kg) ^†^		
Extremely low (<1)	49	1(0, 2)
Very low (1 to <1.5)	280	2(1, 17)
Low (1.5 to <2)	670	8(3, 15)
Low (2 to <2.5)	1448	2(1, 5)
Normal (2.5 to 4)	6253	2(1, 5)
Macrosomia (>4)	352	2(1, 4)

*These are admission episodes. Neonates with multiple diagnoses are counted under each of these making the total admission episodes greater than the study population.

^†^Length of stay values are median (lower, upper quartile).

There were slightly more males (4966/9115, 55%) than females in this population. Most of the neonates (6287/9115, 69%), were of normal birthweight (2.5 to 4 kg) with very few extremely low birthweight category (<1 kg) infants (49/9115, 0.5%). More than one in every three babies admitted were born through caesarean section (3660/9115, 41%). The most common admission diagnosis was birth asphyxia (3867/9115, 42%); however, given the lack of diagnostic facilities there were neonates with multiple diagnoses resulting in total admission episodes greater than the study population (see Table 2 for further details).

The key outcome of interest is in-hospital mortality and almost one in every ten babies admitted died in the unit (839/9115, 9.2%, 95% CI 8.7 to 9.8%). Routine first trimester ultrasound is not available in the Kenyan public sector and estimated gestational age by dates was poorly documented with 70% missing, and is thus not included in Table 2. For the reported clinical characteristics, the highest proportion of observations with missing data was 6% for HIV exposure status.

Data on length of stay and birthweight were available for 9092 out of all the 9115 patients admitted ( Table 2). Most of neonatal admissions have a length of stay less than one week (83%, 7580/9107) with median of 2 days (range 0–98 days). The median stay is less than 5 days in all categories; a reflection of early high mortality and discharge for birthweight <2 kg and >2 kg, respectively. The interquartile range, however, varied, with the widest observed (16 days) for the VLBW. The shortest maximum length of stay was for Macrosomia (33 days) while the longest was for VLBW (98 days).

### Probability of in-hospital mortality or discharge alive

**Figure 1.**
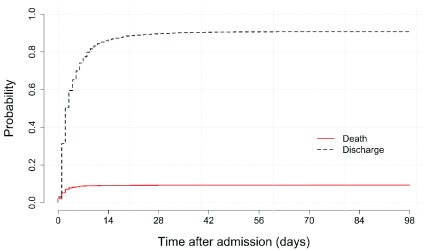
Probability of in-hospital death or discharge alive for all neonates.

***a) Overall.*** Outcome status were available for 9074 out of the 9115 subjects admitted (96%). In-hospital death was observed in 840 neonates, 8126 were discharged alive, and 108 referrals. The longest duration of follow up was 98 days. Figure 1 shows the absolute probability of in-hospital death and discharge alive over the follow-up period.

The probability of being discharged alive rapidly rises and remains higher than the probability of in-hospital death throughout follow up. The probability of in-hospital death peaks out at the overall case fatality rate of 9.2% observed. This peak is attained within the first week after admission, with 22% (188/840, 95% CI 20 to 25%) and 73% (613/840, 95% CI 70 to 76%) of these deaths occurring in the first 24 hours and between 24 hours to 7 days respectively. There was no difference in probability of in-hospitality death and probability of being discharged alive by sex with case fatality rate (CFR) in males 9.1% (449/4944) and 9.6% (391/4089) in females.

**Figure 2.**
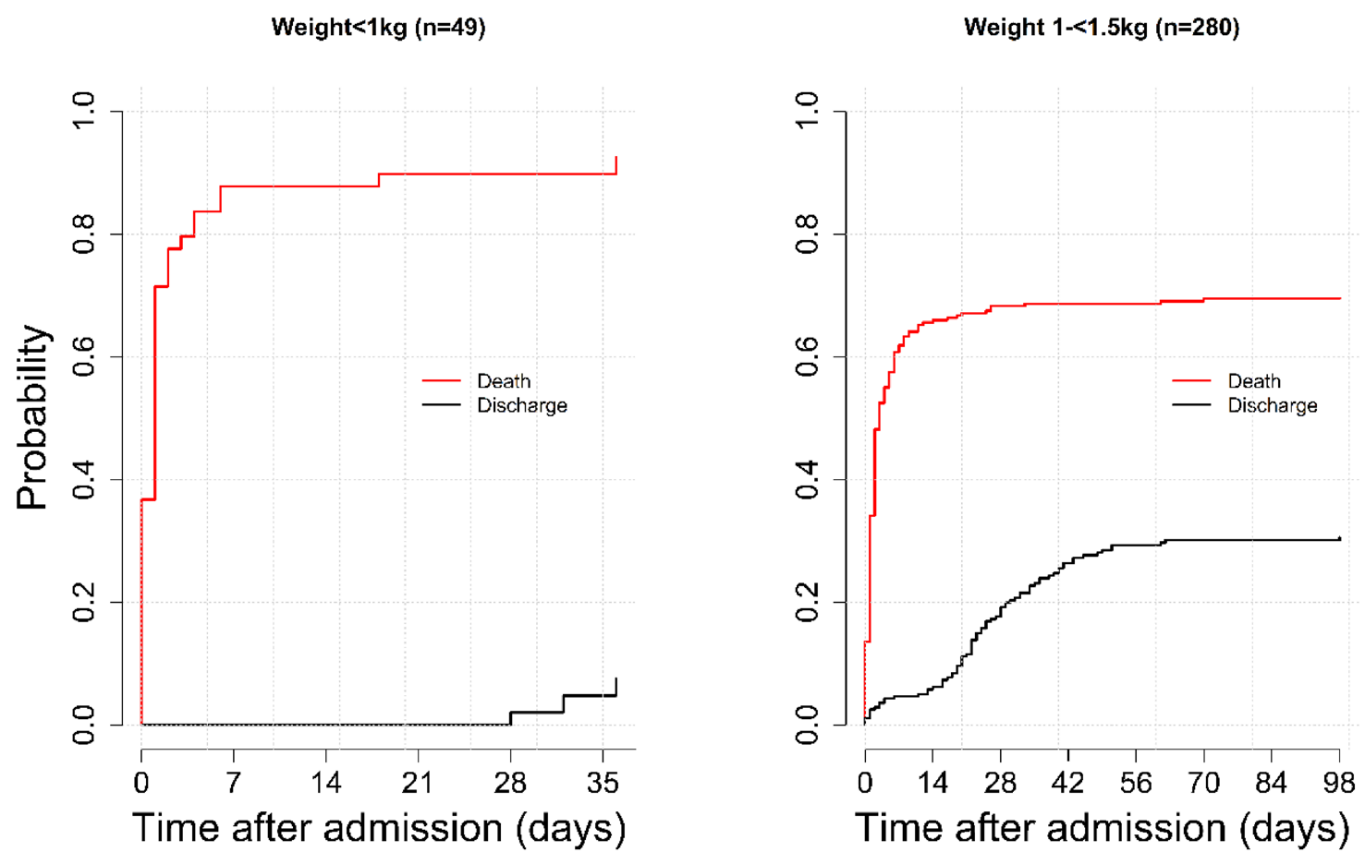
Probability of in-hospital death and discharge alive for birthweight less than 1.5 kg.

***b) By birthweight.*** The probabilities of in-hospital death and discharge alive over time were analysed by birthweight. The lowest categories, i.e. ELBW (<1 kg) and VLBW (1 to <1.5 kg), are shown in Figure 2. In total, 49 babies were born ELBW (45 dead, 1 alive, 3 referred) with CFR of 92% (45/49). There were 280 babies with VLBW, of which 192 died, 79 were discharged alive and nine were referred, and a CFR of 69% (192/280). **

In both groups, there was a steep rise in probability of death in the first day and first week of admission, peaking at >0.9 for ELBW (≈ day 35) and 0.7 for VLBW (≈ day 70). In addition, the probability of death remains higher than the probability of discharge alive throughout the course of hospital stay.

No babies were discharged alive during the first 28 days after admission for ELBW, while there was a gradual rise in the first two weeks for VLBW. The steepest rise in the probability of being discharged alive for VLBW neonates was seen between two weeks (14 days) and eight weeks (56 days) post admission. These difference in patterns of in-hospital deaths and being discharged alive results in the wider gap between the two cumulative incidence curves seen in ELBW as compared to the VLBW.

**Figure 3.**
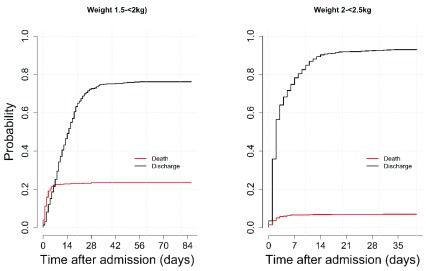
Probability of in-hospital death or discharge alive for birthweight 1.5 to <2 kg and 2 to <2.5 kg.

The definition of LBW category, 1.5 to < 2.5 kg, is widely used in neonatal medicine; in this study, those with LBW comprised 2120 observations (257 dead, 1828 alive, 35 referred), with a CFR of 12% (257/2118) ^31^. The probability of discharge alive was greater than probability of in-hospital death (not shown), in contrast to Figure 2. To investigate this switch in probability of outcome, we split the category into two 500-g groups i.e. 1.5 to <2 kg and 2 to <2.5 kg as illustrated in Figure 3. The two graphs in Figure 3 include 670 neonates in the 1.5 to <2 kg category (157 dead, 493 alive, 20 referred) and 1450 neonates in the 2 to <2.5 kg category (100 dead, alive 1335, referred 15). The case fatality ratio is 23% (157/670) vs 7% (100/1448) for 1.5 to <2 kg and 2 to <2.5 kg categories, respectively.

From Figure 3, it is evident that the switch in survival probability occurs in the 1.5 to <2 kg category around the end of the first week of admission, where we see the cumulative incidence curves crossing. After this point, the probability of discharge alive remains higher than probability of in-hospital death at all time points after admission. The peak probability of risk of in-hospital death rises faster and attains a higher peak in the 1.5 to 2 kg category compared to 2 to <2.5 kg; 23% vs 7%, respectively.

**Figure 4.**
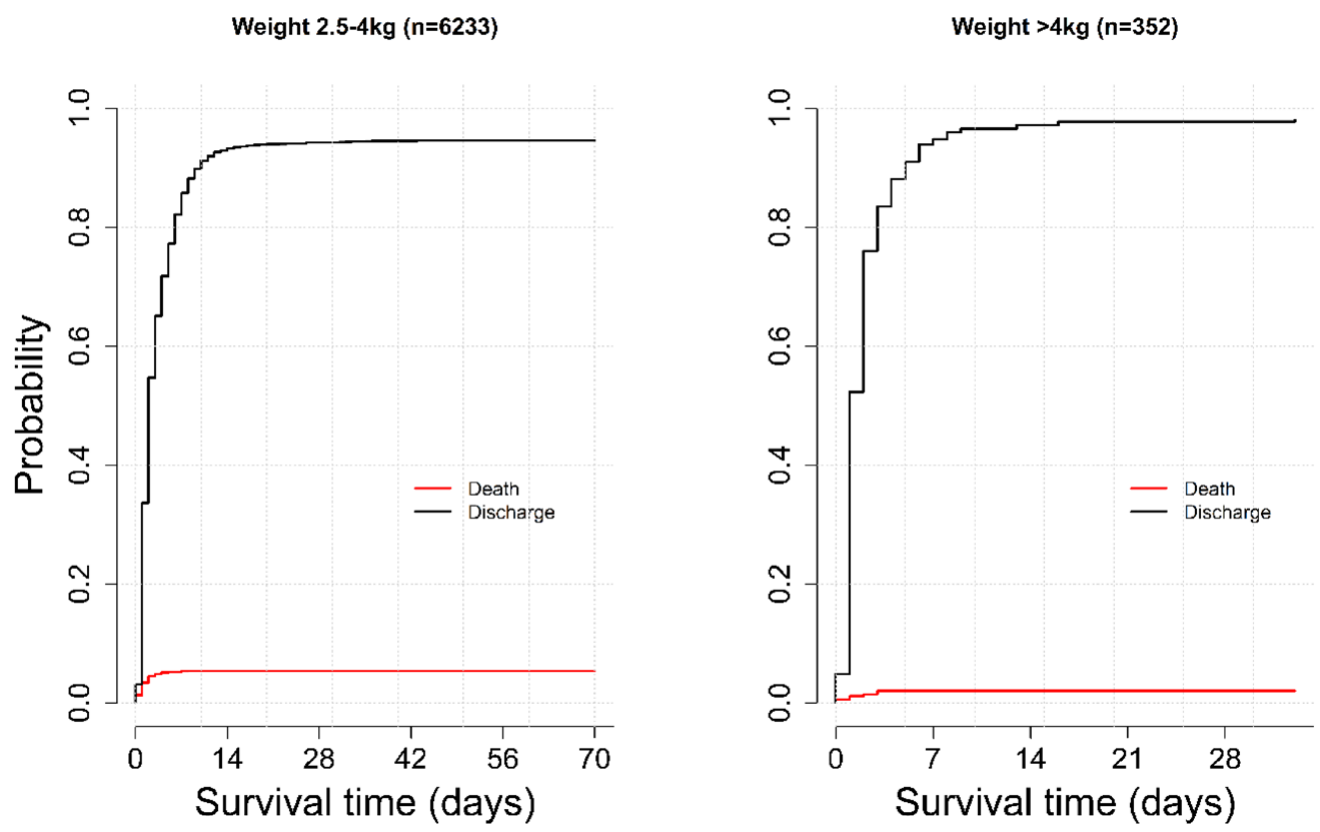
Probability of in-hospital death or discharge alive for birthweight 2.5 to 4 kg and greater than 4 kg.

Figure 4 illustrates survival in the normal weight and macrosomia categories. There were 6233 with normal birth weight (336 dead, 5861 alive, 61 referred) with CFR of 5% (336/6233). The macrosomia category had 352 (7 dead, 343 alive, 2 referred) and a CFR of 2% (7/353).

Figure 4 shows that the maximum length of stay observed in normal weight babies was more than twice that observed in those in the macrosomia category: 70 days vs 33 days, respectively. The lowest estimated absolute probability of in-hospital death is observed in these two categories at less than 0.05.

## Discussion

During the study period spanning a total of 18 months, 9115 neonates were admitted to the neonatal unit. Overall, about 1 in 10 (9%) of neonates admitted died with the highest case fatality among the ELBW (92%) and the lowest among those with macrosomia (2%). Whereas the risk of in-hospital death among the normal birth weight neonates (5%) was about half the overall risk, the absolute number of deaths (336/840 or 40% of all deaths) was the highest, as the majority (70%) of the admissions were of this category. The overall median length of stay was 2 days, with a range of 0–98 days. The ELBW neonates experienced short stays and the highest probability of death in contrast to macrosomic neonates who had short stays but with the lowest probability of death. Our Kenyan data revealed that amongst the traditional LBW category of babies (1.5 to <2.5 kg) that there are considerable differences in outcome between those 1.5 to <2.0 kg and the 2 to less than 2.5 kg sub-category suggesting that neonatal mortality reporting in similar settings should include this sub-categorisation.

The overall CFR in this study (9.2%) lies within the range (3–62%) observed in data available from other Kenyan hospitals ( Table 1) ^22, 24^. However, the extremes of this range represent special populations, i.e. the lowest (3%) was observed in neonatal intensive care in a tertiary private hospital while the highest (62%) included infants with neonatal tetanus only in a rural district hospital ^22, 24^. Nonetheless, 9.2% is low relative to a CFR >10% reported in the majority (12/15) of Kenyan studies ^8, 9, 14– 21, 23– 26, 38^. The CFR in VLBW neonates was 69%, a figure much higher than 45% ^16^ and 44% ^14^ in a Kenyan tertiary hospital and reports of 48% in Malawi ^39^ and 38.9% in Tanzania ^39^. This CFR is, however, similar to the 68.9% reported from a rural NICU in Uganda ^40^. This high risk of death reflects the known association between VLBW and mortality ^41^. This highlights how an overall CFR including all birthweight categories masks significant differences by weight category. Birthweight specific mortality is therefore a useful starting point for cross-site comparison given the availability of these data in settings without gestational age data. It is also clear that it is incorrect to make direct comparisons of mortality between hospitals without adjusting for differences in case mix. Understanding mortality within hospitals nevertheless remains important particularly for understanding and improving the quality of care in addition to tracking change over time ^42, 43^.

Length of stay for most patients (87%) was a week or less. There are few data to compare with from Kenya, with only one study identified ^26^. In this study, Myhre and colleagues reported mean (and range) in days of 39 (10–112) vs 28 ( ^16–54^) before and after introduction of continuous positive airway pressure (CPAP) ^26^. Two studies were identified from other African countries. Pepler *et al.* (South Africa) found that the median was 11 days (range 1–171) while Zash and colleagues (Botswana) found a median of 5 days (interquartile range 2–15) ^44, 45^. If neonatal units in low-resource settings achieve improvements in survival of the admitted neonates, it is anticipated that there will be a concomitant increase in length of stay and resource use. For example, if the CFR rate for VLBW babies in the study facility is reduced from 70% to the 11% seen in the American Vermont Oxford Network, the number surviving to discharge would increase from 79/280 (1817 patient days) to 249/280 (5727 patient days) ^46^. This is a greater than 200% increase in patient days, which would lead to an increase in costs and should be considered in service delivery planning as in-patient care for neonates is developed further in high-mortality settings.

The difference in probability of dying observed after sub-categorization of the LBW ( Figure 3) suggests the need for more granular weight such as 100- or 250-g intervals in reports from neonatal networks in high-income countries ^47, 48^. There were no other instances of competing risk analysis in neonatal care pertaining to hospitals in Kenya or other African countries identified in the period 2007–2017. Hinchliffe *et al*. computed the probability of dying, being discharged and being cared for in the unit over time for 29 neonatal units in USA. They included neonates of gestational age 24–28 weeks admitted over a 5-year period (January 2006 to December 2010) ^12^. Two key similarities with our study are observed. Firstly, the highest rates of in-hospital death occur in the initial weeks after admission. Secondly, the probability of survival to discharge rose with increase in birthweight ^12^.

The competing risk method provides an accurate estimate of the probability of in-hospital mortality in the presence of a mutually exclusive alternate outcome (discharge alive) ^10, 11^. This is achieved by simultaneously estimating the probability of in-hospital mortality and discharge alive, in contrast to the Kaplan-Meier method where the competing outcome is right-censored leading to overestimation of probability of the outcome of interest ^10, 11^. Competing risk analyses as demonstrated in this study could thus be useful in communicating the probable length of stay by birthweight category to clinicians caring for neonates in high-mortality, low-resource settings. Such information can also be useful in communicating to parents on when they are likely to leave for home ^12^. In addition, in examining variation between units, such estimates would allow a more detailed comparison of patients than that afforded by overall case fatality rates only. Length of stay as a key determinant of hospital costs is useful in projecting resource requirements to inform resource allocation. With the anticipated improvement in survival to discharge, particularly for those with VLBW and ELBW, the resultant long hospital stays mean accurate estimates of probability of discharge at different time points, as provided by competing risk analyses, will be necessary to inform service delivery planning and organisation.

## Limitations

These data are from one site and therefore represent only one context within a low resource setting. This facility has limited capacity to offer more advanced respiratory support (BCPAP) with only two machines, while many public sector neonatal units in Kenya have even more limited capacity. However, the facility has in common with other low-resource settings the provision of essential neonatal care with resource constraints particularly low nurse to patient ratios ^49^. Gestational age is a key risk factor for neonatal outcomes but these data were largely missing. The lack of gestational age data means small for gestational age neonates are not identified in the context of low- and middle-income countries (LMICs),where the burden is estimated to be very high (32.4 million in 2010) ^50^. In-hospital mortality may be subject to bias as there may be variation in discharge criteria particularly when discharge is followed by unobserved early post discharge mortality. However, these data are useful for informing decision making particularly for groups of patients and for service planning.

## Conclusion

Case fatality, length of stay and time to in-hospital death are important outcomes with respect to fundamental prognosis in neonatal hospital care. The observed difference in length of stay for the ELBW and VLBW babies overall and those discharged alive suggests that improved survival for these neonates will lead to longer hospital stays with attendant increase in costs requiring planning for this scenario. Attention also needs to be paid to the normal weight babies with regards to reduction of absolute numbers of deaths. The competing risk analyses provided estimates of cumulative incidence of in-hospital mortality, the probability of discharge alive over time and demonstrated residual variation in risk of death in birthweight categories. This residual variation suggests that better methods of estimating risk of in-hospital mortality, particularly at individual patient level are required. Data from this study are likely to be applicable to other district hospital level neonatal units in LMICs where intensive care is not available as evidenced by similarities in the case fatality rate. Nevertheless, more of such data are required from other neonatal units in Kenya and other low resources settings (akin to neonatal clinical networks in high income countries such as the Canadian Neonatal Network) to support service improvement and monitoring especially as more advanced neonatal care is scaled up ^48^.

## Data availability

### Underlying data

The source data are owned by the Kenyan Ministry of Health, County Governments and as the data might be used to de-identify hospitals the study authors are not permitted to share the source data directly. Users who wish to reuse the source data can make a request initially through the KEMRI-Wellcome Trust Research Programme data governance committee. This committee will supply contact information for the KEMRI Scientific and Ethical Review unit, County Governments and individual hospitals as appropriate. The KEMRI-Wellcome Trust Research Programme data governance committee can be contacted at: dgc@kemri-wellcome.org.
