# Supplementary material for: Competing risk survival analysis of time to in-hospital death or discharge in a large urban neonatal unit in Kenya
Source: Wellcome Open Res. 2019 Jun 17;4:96. [Version 1] doi: 10.12688/wellcomeopenres.15302.1 (PMC6611136; doi:10.12688/wellcomeopenres.15302.1)
Supplement: Peer review history

# Peer review: Reviewer response for version 1

**DOI:** 10.21956/wellcomeopenres.16702.r35786

**Published:** 2019-08-07

**Authors:** Cheryl Battersby

**Affiliations:** 1 Section of Neonatal Medicine, Imperial College London, London, UK

**General comments**

 This is an important and well written manuscript. I commend the authors for addressing a major problem for newborn care in low resource settings – the paucity of data to evaluate health care systems. Data collection and analysis are often neglected in low resource settings because of the lack manpower. However, it is in resource-poor environments that data are ever more crucial to inform the allocation of the limited resources to bring about the biggest improvements in care and outcomes. This study demonstrates feasibility of acquiring maternity and neonatal data in low-income countries and is an exemplar for other countries.

**Overall findings**

 This is a single centre study in Nairobi, providing important base-line data on mortality rates by birth-weight category for neonates admitted to a busy neonatal unit. 9,115 infants were admitted over an 18-month period April 2014-Dec 2015. Most babies were admitted for a short period of time (median 2 days) and most deaths occurred early in the first week of life.

 There were considerable differences in outcomes for babies in different birth weight categories. Mortality among  <1kg was 92%, (45 babies), 1 to <1.5kg 69% (192 babies), 1.5 to <2 kg 23% (157 babies), 2 to <2.5kg 7% (100 babies), normal (2.5 to 4kg) 5% (336 babies), macrosomia (>4kg) 2% (7 babies).

 Although the range 1.5 to <2.5kg is usually classified as low birthweight, the authors suggest other studies in similar settings should sub-categorise into 1.5 to <2kg (23% mortality) and 2 to <2.5kg (7% mortality) because of the marked differences in mortality rates.

**General areas to consider  **

1. I would suggest the abstract highlight some of the findings mentioned above.
2. The caesarean section rate amongst those admitted was 41%; how does this figure compare with the overall caesarean section rate? It would also be useful to clarify the admission criteria because in my experience, in some low-income countries, all babies delivered by caesarean sections are admitted for a period on the neonatal unit.
3. I suggest the discussion can provide a more in-depth narrative on the utility of these data; to suggest strategies to improve outcomes considering the results. For example, the proportion of babies with birth asphyxia (42%) suggests that resources need to focus on improving perinatal care/obstetric management.

**Specific areas to consider**

**Abstract:** There is a typo in results – should read 4966/9115 for males.

**Introduction: **A well written introduction, highlighting the gaps in data and inconsistency in reporting. Useful to include the 15 published reports from Kenya in the last 10 years, but suggest to highlight that the case fatality is not always among those admitted to neonatal units, but are subgroups e.g. patients with blood cultures positive sepsis only, and neonatal tetanus. This is provided in the legend below the table but would be good to include in the text. Please check consistencies in the use of symbols in table and legend below it; I think the symbol for *same facility, different studies *should be two crosses on top of each other rather than one.

**Results:**

 In results, second paragraph it reads “ *however, given the lack of diagnostic facilities there were neonates with multiple diagnoses resulting in total admission episodes greater than the study population”*. Please can you clarify what this means?

 It is normal for a baby to have more than one diagnosis; I also think the use of the term ‘admission episodes’ causes confusion. An *episode* in many neonatal databases usually represents a discrete period of care in a single neonatal unit.

 I also think the text under corresponding Table 2 can be simplified. I suggest changing text from the current “*…. *Neonates with multiple diagnoses are counted under each of these making the total admission episodes greater than the study population*” to simply “Neonates can have more than one diagnosis”.

**Please clarify some of the numbers in the Results section:**

 In paragraph 4 “ *Data on length of stay were available for 9092 out of all the 9115 patients admitted (Table 2)” . *However Table 2 for length of stay total is 9052 not 9092. Is there a typo? 

 In the paragraph that begins with *“Probability of in-hospital mortality or discharge alive a) overall*”, it reads “O *utcome status were available for 9074 out of the 9115 subjects (96%)”; *but this is 99% not 96%. Then it provides numbers for in-hospital deaths (840), alive (8126) but these do not agree with Table 2 which reads 839 and 8135, respectively. Table 2 shows that there are outcomes for 9082 not 9074; (8135 (discharge alive)+839 (dead)+108 (Referred) = 9082 (33 have missing)). Can these numbers please be clarified?

**Figures: **Suggest use dashed and smoothed lines as in Figure 1 (rather than colour) for all survival curves. This would make it practical for those printing in black and white in low resource settings.

I confirm that I have read this submission and believe that I have an appropriate level of expertise to confirm that it is of an acceptable scientific standard.

# Peer review: Reviewer response for version 1

**DOI:** 10.21956/wellcomeopenres.16702.r35788

**Published:** 2019-07-04

**Authors:** Alison A. Leaf

**Affiliations:** 1 Department of Child Health, University Hospital Southampton NHS Foundation Trust, Southampton, UK

This is a clear and timely article providing important base-line data on mortality rates by birth-weight category for neonates admitted to a single, large, busy neonatal unit in a maternity hospital in Nairobi. The introduction highlights the fact that there is no system for consistent and accurate data collection of this type in Kenya and this study provides a potential model.

 Data were collected at time of discharge, death or referral for 9115 babies admitted during an 18 month period and analysed retrospectively. Analysis was done using the method of 'competing risk survival analysis' where the 'competing risks' were of death OR discharge alive. This is helpful, as these outcomes are unambiguous and clear, and there is a consistent denominator in the total number of admissions. A small number of babies were referred elsewhere, and thus their final survival category is unknown, but this was just over 1% of total, so does not significantly alter the key messages.

 The clinical variables collected were: sex, birth weight, mode of delivery, place of delivery, admission diagnosis and outcome; birth weight  was divided into 5 standard categories - ELBW (<1 kg), VLBW (1-1.5 kg), LBW (1.5-2 kg), Normal weight (2.5-4 kg) and Macrosomia (>4 kg). The top three diagnoses were birth asphyxia (42%), preterm/LBW (22%) and neonatal sepsis (11%).

 Overall mortality rate was 9.2% and, not surprisingly, this was highest in the ELBW and VLBW infants (92% and 69% respectively). Most deaths occurred between 0-24 hours (22%) and 24 hours-7 days (73%) after admission, and the survival curve for mortality was particularly steep in ELBW infants. There was a slower rise and narrower gap in VLBW, and as the authors describe, these graphs can give a clear illustration of how lengths of hospital stay, and need for increased resources, would rise as mortality rates fall in the future.

 Data for the LBW group were subdivided and revealed significant differences between infants born at 1.5 to 2. 0 kg and 2.0 to 2.5 kg. This gives important insight into where specific improvement initiatives should be targeted. The highest number of deaths (336) was in the 'normal' birth weight group. This group, however, covers a wide range for birth weights - from 2.5 to 4.0 kg. Analysing this data in 500 g sub-groups would probably also reveal differences which could focus improvement initiatives. Reference is made to some of the well-established neonatal data bases, such as Vermont Oxford, UK and Canadian, and over time lessons can be learned from those as more sophisticated data collection and analysis becomes possible.

 The strengths of this study are its size (almost 10,000 cases), its completeness (over 99% birth weight and length of stay) and its simplicity. The limitations are well covered in the paper and include single site, lack of gestational age data (and thus inability to identify proportion of IUGR infants) and potential for inaccuracy in main diagnosis. Despite this the database provides a robust and useful tool against which progress can be monitored both within this centre and between comparable neonatal units.

 Areas to consider for improvement:

1. High rate of caesarean section - is this reflective of overall C/s rate, or are more C/s babies admitted to NBU?
2. High incidence of perinatal asphyxia - as only 3% of admissions were outborn, it is hoped that combined maternity and neonatal quality improvement programs could address this within the hospital?
3. If sharing data with other units it would be important to adjust for resources available - both human resources as in medical and nursing staff, and technology/equipment.
4. Improving survival in each of the different weight categories will require somewhat different strategies - for example ELBW and VLBW infants often need more technological support. However, for all weight categories important work can be done to ensure 'essential newborn care' is optimised, with attention to hand and environmental hygiene, lactation support, Kangaroo Mother care, antibiotic stewardship etc. On-going data collection can help to show the impact of such interventions, creating a positive feedback cycle for staff.
5. Analysing the 'normal birth weight' group in 500g sub-groups might reveal important differences.
6. As commented by the previous reviewer, some information on discharge and referral criteria would also be helpful.
7. One small type error noted: in the Abstract the ratio of male patients is written as 966/9115, and should be 4966/9115

I confirm that I have read this submission and believe that I have an appropriate level of expertise to confirm that it is of an acceptable scientific standard.

# Peer review: Reviewer response for version 1

**DOI:** 10.21956/wellcomeopenres.16702.r35787

**Published:** 2019-07-01

**Authors:** Elizabeth Molyneux

**Affiliations:** 1 Department of Paediatrics, University of Malawi, Blantyre, Malawi

This is a retrospective cohort study looking at routine data collected on all the admissions to a busy public hospital neonatal unit. Data are entered in real time, on discharge, death or referral of a baby from the hospital. The study period covers 18 months from April 2014 – December 2015, though there were no admissions from April to June 2015 because of health staff strikes.

 The data collected included sex, birth weight, mode of delivery, place of delivery, admission diagnosis, length of stay and outcome (death, discharged alive or referred to another hospital). The DHIS2 dataset, routinely collected for national reporting, was insufficiently detailed and poorly collected. The study data were collected on the clinical information network (CIN) database on which more detail is recorded and the quality of data entry is monitored.

 The hospital is busy, the neonatal unit admitted 9115 newborns during the study period, despite not functioning for 3 months. The annual admissions were 4,500 with 60 babies in the unit at any time being looked after by 2-3 nurses per shift. The most common diagnosis for admission was birth asphyxia (42%) and 1/3 of all babies were born by Cesarean section. Essential newborn care was provided with the ability to give CPAP to some babies. Four paediatricians, 6 medical officers and 4 clinical officers gave 24 hour medical cover.

 Of the study group, just over half were male (55%) and 69% were of normal (2.5-<4 kg) birth weight. The median length of stay was 2 days and the overall mortality 9.2%. 22% of deaths occurred in the first 24 hours of life and 73% in the first week of life.

 The data were analysed using a survival analysis approach to outcome using length of stay and birth weight which are competing risks for survival.

 In this report we are given the outcomes and lengths of stay in the unit for newborns in different weight categories ELBW (<1 kg); VLBW (1-< 1.5kg); LBW 1.5kg - <2.5kg), normal birth weights (2.5 -<4 kg) and macrosomia >4kg. Unsurprisingly the extremely low birth weight babies have the earliest and highest mortality (92% case fatality rate CFR) with only one surviving longer than 4 weeks and the probability of death remaining high during the entire hospital stay. The VLBW babies had a CFR 69% with longer length of stay and as admission weights increased, mortality decreased and the risk of death over time decreased. LBW infants had a CFR of 12%  and normal infants of 5% and macrosomia of 2%.

 Interestingly when the LBW group were subdivided into those between 1.5 - <2 kg and 2 -<2.5kg the outcomes were very different with 23% mortality in the former and 7% in the later.

 The other useful data are the number of referrals by birth weight. There were only 49 ELBW babies of whom 3 were referred; Of 280 VLBW babies, 9 were referred; 670 babies were 1- < 1.5 kg of whom 20 were referred; 1450 babies were 1.5 - <2 kg and 15 were referred, of the 6233 normal birth weight babies 61 were referred and 2 of the 352 macrosomia infants.

 The authors have done these analyses to make better inter-hospital comparisons but also to try to inform decisions on the organisation of neonatal services.

 The authors acknowledge the limitations when a pragmatic study is embedded in usual normal clinical care including that diagnoses may not have been correct. 

 It would be useful to know what are the discharge criteria, the referral criteria and are birth weights accurate?

 Did the data include babies in a kangaroo mother care unit?

 The LSCS rate seems very high (41%) and the birth asphyxia very common. If this diagnosis is correct much of the ability to make change lies with the obstetric department.

 How many, especially of the ELBW babies had congenital abnormalities, how many had surgical conditions meriting long hospitalisation?

 Is there any way of identifying nosocomial infections in the long stay infants?

 This is a well written, clear and very interesting paper. It has provided data to confirm what many clinicians know about mortality and its relationship to birth weight, but using that data to inform the health systems and its reorganisation is important. With the drive to reduce neonatal mortality and improve the quality of care in LMICs, survival will improve but hospital stays will be longer and treatment more complex. Unless thought, driven by evidence such as this, is given to space, staffing, equipment and training any improvements will be held back.

I confirm that I have read this submission and believe that I have an appropriate level of expertise to confirm that it is of an acceptable scientific standard.
